# P03.07. Thematic analysis of resident application essays for a nutrition education program

**DOI:** 10.1186/1472-6882-12-S1-P260

**Published:** 2012-06-12

**Authors:** R Graham, C Ngamwajasat

**Affiliations:** 1 Lenox Hill Hospital, New York City, USA

## Purpose

Obesity is on the rise in the United States and there has been a conscious effort on the part of the government and physicians to provide measures to counteract the epidemic. Diet is an important part of therapeutic lifestyle modification, but many physicians feel lacking in their ability to provide sound and adequate counseling. Our objective was to assess the main reasons why residents at Lenox Hill Hospital chose to enroll in a nutrition education program.

## Methods

Sixteen residents of varying specialties at Lenox Hill Hospital wrote essays to apply to a nutrition education program given by the hospital. A theme analysis was applied to assess common reasons why residents chose to apply to the program. Each theme was assigned a letter and the number of appearances of a theme was tabulated.

## Results

There were seven main themes that were consistently present in the residents’ essays as to why they wanted to enroll in a nutrition education program: 56% alluded to a lack of nutrition education; 44% listed diseases with relation to diet; 44% wrote about a desire to make changes to their own lives; 31% spoke about improving patient health outcomes; 25% spoke about the obesity epidemic; 19% about wanting to “practice what they preach” in terms of nutrition; and 13% thought the program could help them field patients' questions about nutrition.

## Conclusion

Young physicians at Lenox Hill Hospital chose to apply to a nutrition education program because of a recognition of a disease epidemic related to diet, a desire to improve personal and patient education and outcomes, and above all, the perception that they lack sufficient nutrition education.

